# The Co-Occurrence of Medullary and Papillary Thyroid Carcinoma—A Literature Review Based on a Case Report

**DOI:** 10.1155/2024/2393186

**Published:** 2024-10-17

**Authors:** Katharina Nordhausen, Timo Deutschbein, Volker Heinrichs, Dirk Weyhe, Navid Tabriz

**Affiliations:** ^1^University Hospital for Visceral Surgery, Pius-Hospital Oldenburg, Carl Von Ossietzky University Oldenburg, Georgstr. 12, Oldenburg 26121, Germany; ^2^Medicover Oldenburg MVZ, Elisenstraße 12, Oldenburg 26122, Germany; ^3^Department of Internal Medicine I, Division of Endocrinology and Diabetes, University Hospital, University of Würzburg, Josef-Schneider-Straße 2, Würzburg 97080, Germany; ^4^Division of Nuclear Medicine, Pius-Hospital Oldenburg, Carl Von Ossietzky University Oldenburg, Georgstr. 12, Oldenburg 26121, Germany

## Abstract

**Background:** The co-occurrence of medullary thyroid carcinoma (MTC) and papillary thyroid carcinoma (PTC) is thought to be a rare phenomenon. Here, we present a patient undergoing surgery due to a suspected MTC. Histopathological workup confirmed the suspected diagnosis but also described an incidental PTC in the contralateral lobe with lymph node metastasis in the central cervical compartment.

**Case Presentation:** A 58-year-old female presented with thyroid nodules and significantly elevated levels of calcitonin and carcinoembryonic antigen (CEA). She underwent total thyroidectomy along with central and bilateral lymph node dissection. Histology revealed a MTC of 20 mm and a BRAF V600E-positive PTC of 11 mm with central cervical lymph node metastasis. Postoperatively, ablative radioiodine therapy was performed. Two months later, both calcitonin and CEA were normalized.

**Conclusion:** Simultaneous occurrence of MTC and PTC seems to be rare, but recent literature suggests that simultaneous occurrence is probably more frequent than initially thought. Preoperative calcitonin can be helpful in the diagnostic workup of thyroid nodules. Due to different treatment strategies, precise histological differentiation of potential lymph node metastasis is essential.

## 1. Introduction

In recent years, there has been a worldwide increase in mainly differentiated thyroid carcinomas with a constant mortality rate, which is most likely due to improved diagnostic and therapeutic means [1]. Thyroid cancer can be divided into different subtypes, which differ significantly in terms of their metastatic pathways, treatment options, and prognosis [2]. Papillary thyroid carcinoma (PTC) is responsible for around 80% of all differentiated thyroid tumors [3]. They originate from cells of the follicular epithelium [4], and exposure to ionized radiation as well as Hashimoto's thyroiditis has been described as potential risk factors [5]. Medullary thyroid carcinoma (MTC) is much rarer, accounting for around 2%–4% of all thyroid malignancies [3, 6]. Arising from parafollicular endocrine cells, they excessively secrete the peptide hormone calcitonin. Tumor occurrence may be associated with the presence of multiple endocrine neoplasia type 2 [6].

Since its first description by Lamberg et al. [7] in 1981 [7], in addition to individual case reports, at least nine retrospective case series on the simultaneous occurrence of PTC and MTC were published, with cohort sizes ranging from 5 to 183 patients (340 patients in total) [8–16]. Here, we report a case with concomitant PTC and MTC, illustrating the common diagnostic and therapeutic workup. Furthermore, we provide a literature review to allow for a better estimation of the true prevalence of simultaneous thyroid cancer of different origin.

## 2. Case Presentation

**Figure 1:**
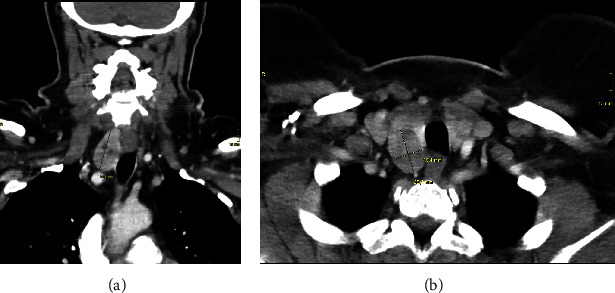
Preoperative computed tomography scan of the neck. (A) Computed tomography scan with the right thyroid lobe enlarged dorsally but without evidence of cervical lymph node metastasis. (B) The right thyroid lobe is increased to 18 mm × 25 mm × 33 mm in the dorsal part (dimensions are highlighted with solid lines).

A 58-year-old female with known asymptomatic thyroid nodules was referred for a yearly routine follow-up investigation. There was no prior exposure to radiation therapy, and the family history was negative for endocrine diseases (particularly for thyroid cancer or multiple endocrine neoplasia type 2 just as autoimmune disorders of the thyroid). Ultrasound showed a thyroid gland of normal size (14 mL) with a progressive isodense nodule of 18 mm × 17 mm × 14 mm in the right central lobe, European Thyroid Imaging and Reporting Data System (EU-TIRADS 3), and a small hypoechoic ventrobasal nodule of 7 mm×6 mm×6 mm in the left lobe, EU-TIRADS 4, so that further diagnostics were initiated. According to 55 MBq Tc99m pertechnetate scintigraphy, currently routinely carried out in Germany for diagnosis of thyroid nodules, the uptake of 2.12% was normal and homogenous. Fine-needle aspiration (FNA) of the right-sided thyroid nodule revealed a follicular neoplasia (Bethesda IV), which was consistent with MTC according to an expression of calcitonin in immunohistochemistry. The nodule on the left side was not investigated via FNA. Laboratory values determined on the basis of the suspicion of MTC showed highly elevated levels of calcitonin (537.3 pmol/L, normal range ≤ 1.72 pmol/L) and carcinoembryonic antigen (CEA) (31.1 ng/mL, normal range ≤ 2.5 ng/mL), without the use of proton-pump inhibitors, along with euthyroidism. A preoperative computed tomography scan of the neck, thorax, and abdomen did not show evidence of conspicuous cervical lymph nodes or distant metastases (Figure 1).

**Figure 2:**
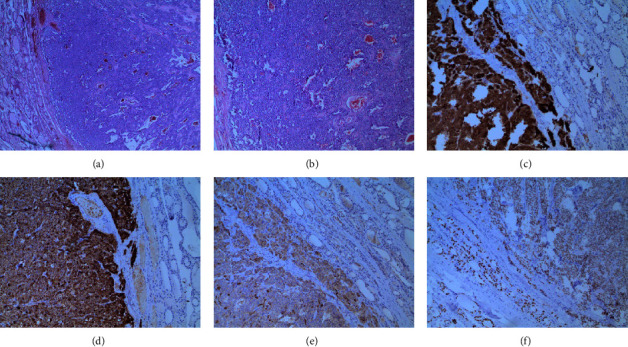
Staining of the right-sided medullary thyroid carcinoma: (A) low-power (40x) and (B) high-power (100x) H&E stain, (C) high-power (100x) CEA stain, (D) high-power (100x) chromogranin A stain, (E) high-power (100x) calcitonin stain, and (F) high-power (100x) TTF-1 stain. CEA, carcinoembryonic antigen; H&E, hematoxylin and eosin; TTF-1, thyroid transcription factor 1.

Due to the high suspicion of MTC and highly elevated levels of calcitonin, after a detailed explanatory discussion, the patient underwent total thyroidectomy with central (level VI) and lateral (level II–IV) lymph node dissection. Histological examination revealed a tumor (with a maximum diameter of 20 mm) in the right thyroid lobe (moderately positive for thyroid transcription factor 1 [TTF-1] and a predominantly negative for paired-box-protein 8 [PAX-8]). Included follicles showed thyrocytes with clearly positive signals for nuclear PAX-8 and cytoplasmic chromogranin A, a cytoplasmic coexpression of the epithelial intermediate filament CK7, and positive reaction for the oncoprotein cyclin D1 (Figure 2). Taken together, these findings confirmed presence of MTC (pT1b).

**Figure 3:**
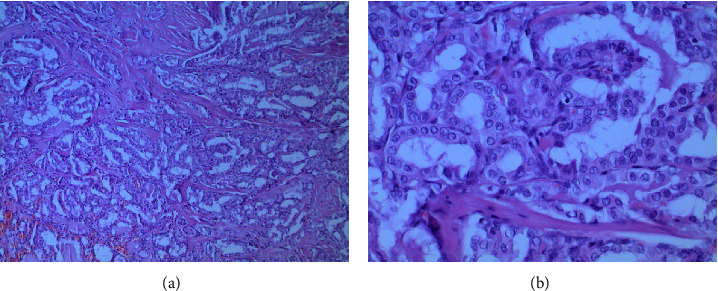
Staining of the left-sided papillary thyroid carcinoma: (A) low-power (40x) and (B) high-power (100x) H&E stain. H&E, hematoxylin and eosin.

Surprisingly, a PTC of 11 mm without infiltration of the capsular boundary and the parathyroid tissue was detected in the left thyroid lobe (Figure 3). In the resected lymph nodes, one 5-mm lymph node metastasis of the PTC (pT1b and pN1a) without infiltration of the perinodal tissue was found in level VI or the central compartment I according to Dralle [17]. Genetic workup via parallel sequencing revealed an activating mutation in the BRAF gene (V600E), whereas other genes (KRAS, NRAS, and RET) showed the unmutated wild type.

**Figure 4:**
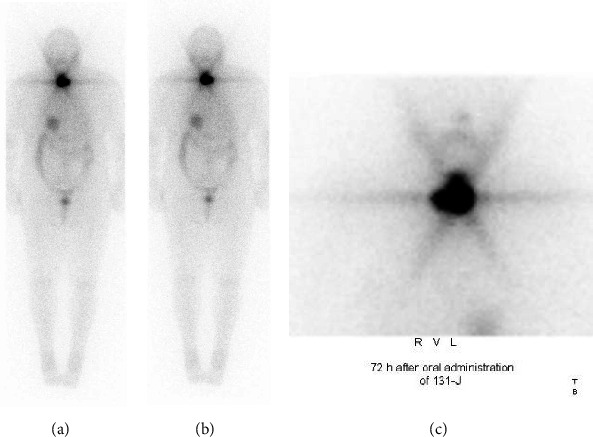
Whole-body scans 72 h after oral administration of 3000 MBq 131-J. From the anterior (A) and posterior (B) as well as the static view of the neck and upper mediastinum (C) showing tracer accumulation in the thyroid lodge as an indicator of postoperative thyroid remnants. No extrathyroidal tracer accumulation is seen.

Postoperatively, no complications were observed, and the patient was discharged on the third postoperative day. Fifty-two days after surgery, a 131-J scintigraphy was conducted. In Figure 4, tracer accumulation in the thyroid lodge 72 h after oral administration of 3000 MBq 131-J is shown, indicating postoperative thyroid remnants. Accordingly, ablative radioiodine therapy was performed by oral application of 3,000 MBq iodine131 in a state of biochemically overt hypothyroidism (i.e., thyroid-stimulating hormone [TSH] > 100 µIU/mL, normal range 0.27–4.20 µIU/mL) 2 months after surgery. Two months later, both calcitonin (0.33 pmol/L) and CEA (0.8 ng/mL) were normalized.

## 3. Discussion

Simultaneous occurrence of PTC and MTC is usually a coincidental phenomenon. A review of the English literature revealed 43 publications on the simultaneous occurrence of PTC and MTC. Of these, 34 publications were individual case reports, and 9 publications described larger case series. These are listed in Table 1. Machens and Dralle reported a significantly smaller tumor mass if MTC and PTC occurred simultaneously, compared to isolated MTC (11 mm vs. 20 mm, *p*=0.008) and PTC (8 mm vs. 20 mm, *p*=0.001) [10]. In 82% of all cases listed with concomitant tumors, the PTC was a microcarcinoma. In all case series, except the one published by Zhang et al. [16], lymph node metastases were more frequently assigned to the MTC, and women were affected more frequently (Table 1).

In our patient, however, the lymph node metastasis was assigned to the BRAF positive PTC of 11 mm. A BRAF mutation can be detected in approximately 45% of all patients with histologically confirmed PTC [18] and is associated with a more aggressive disease course, which includes presence of extrathyroidal infiltration, lymph node metastasis, loss of radioiodine avidity, and higher probability of recurrent disease [19–21]. Furthermore, it has been shown that a BRAF mutation is significantly associated with increased carcinoma-related mortality [22]. In the case series provided in Table 1, the BRAF mutational status of the PTC was only reported by Ciampi et al. [11], with only one out of 24 patients (4.2%) harbored the V600E BRAF mutation. Since BRAF mutation is highly attributed to cancer [23], knowledge of the mutational status is certainly helpful when deciding on the degree of lymph node dissection.

Out of the 26 cases with both tumor entities reported in the study by Machens and Dralle, only one was already diagnosed preoperatively. In the other 25 cases, the second carcinoma (PTC, *n* = 23; MTC, *n* = 2) was an incidental finding during histological examination [10], similar to the case series by Zhang et al. [16], in which only in two cases (9.4%) both tumors were detected preoperatively [16]. Neither in the reported cases by Biscolla et al. [8], Kim et al. [9], and Ciampi et al. [11] nor by Appetecchia et al. [12] were both tumors diagnosed preoperatively in any case. In each case, surgery was performed due to evidence of one tumorigenicity, mostly MTC, or for other reasons with postoperative incidental findings of both PTC and MTC.

This is similar to our current case report. Of note, MTC are therefore obviously not only the leading tumor subtype at initial diagnosis of thyroid cancer but also the tumor subtype which mainly predicts prognosis [12].

In case of a MTC, currently, complete thyroidectomy with central lymph node dissection or additional lateral lymph node dissection (in case of suspected lateral lymph node metastases on imaging) is the treatment of choice [24, 25]. According to established guidelines, preoperative measurement of calcitonin is recommended in case of newly diagnosed thyroid nodules [24, 26]. If calcitonin is clearly elevated, further diagnostics (e.g., FNA) is not regarded as mandatory before surgery. In our patient, however, the decision for fine needle aspiration was driven by progressively increasing nodules in the right thyroid lobe, as calcitonin was not part of the initial diagnostic workup. It remains unclear whether preoperative FNA of the nodule on the left side with BRAF determination would have confirmed the diagnosis of PTC preoperatively. Nevertheless, due to the highly elevated level of calcitonin (1836.3 pg/mL), a thyroidectomy was indicated [24]. Importantly, the indication for surgery should be made separately for each half of the thyroid [26].

Determination of calcitonin already at the time of the initial diagnosis of thyroid nodules might have led to an earlier diagnosis of MTC, thereby potentially reducing the operative risk for complications due to more radical resection. Therefore, we would like to emphasize the value of calcitonin determination in the general diagnosis of thyroid nodules, as it influences a possible surgical strategy.

Especially since FNA of MTC can lead to misdiagnosis, which is described in the literature in up to 50% of cases [27]. As in our case, a follicular neoplasia (Bethesda IV) was described, which results from the presence of cells with round nuclei of various sizes and no typical particles nor a well-defined margin of the cytoplasm [28].

## 4. Conclusion

The number of reported cases with simultaneous PTC and MTC is increasing, most likely due to relevant improvements in preoperative screening and histological workup over time. We here present a case report and a review of literature with concomitant PTC and MTC, highlighting the importance of early routine calcitonin measurement in the presence of thyroid nodules, as its increase influences the indication and extension of the operative strategy. In case of carcinoma PTC, genetic workup especially for a BRAF V600E mutation may pave the way to an optimized degree of thyroid and lymph node resection.

## Figures and Tables

**Table 1:** Literature review of nine case series reporting simultaneous occurrence of PTC and MTC.

Publication (author, year)	No. of cases with simultaneous MTC + PTC	Females (*n*, %)	Mean MTC tumor diameter (mm)	Micro-PTC (*n*, %)	Origin of cervical lymph node metastasis			
					None	MTC only	PTC only	MTC + PTC
Biscolla et al. [8]	27	18 (66.7%)	NA	21 (77.8%)	12^a^	9^a^	5^a^	-^a^
Kim et al. [9]	10	9 (90%)	29 (5–56)	10 (100%)	6	2	1	1
Machens et al. [10]	26	14 (53.8%)	11 (7–15)	NA	13	6	2	5
Ciampi et al. [11]	24	18 (75%)	14 (1–50)	19 (79.2%)	18	6	0	0
Appetecchia et al. [12]	183	105 (57.4%)	NA	148^b^ (83.6%)	NA^c^	52^c^	23^c^	NA^c^
Thomas et al. [13]	21	11 (50%)	31 (9–75)	18 (85.7%)	5	12	2	2
Fallahi et al. [14]	5	2 (40%)	19 (2–47)	4 (80%)	3	2	0	0
Akgun et al. [15]	14	8 (57.1%)	25	11 (78.6%)	5	8	1	0
Zhang et al. [16]	30	13 (43.3%)	16 (1–110)	26 (86.7%)	15	4	9	2

Abbreviations: F, female; M, male; MTC, medullary thyroid carcinoma; NA, not available; PTC, papillary thyroid carcinoma.

^a^One case with unknown tumor staging.

^b^Six cases with unknown tumor size.

^c^The affection of lymph nodes was not further elucidated (i.e., no lymph node metastasis at all, lymph node metastases of one tumor identity only, or simultaneous lymph node metastases of both tumors).

## Data Availability

All data generated or analyzed during this study are included in this published article.

## Nomenclature

CEA: Carcinoembryonic antigen
EU-TIRADS: European Thyroid Imaging and Reporting Data System
FNA: Fine-needle aspiration
MTC: Medullary thyroid carcinoma
PTC: Papillary thyroid carcinoma
TSH: Thyroid-stimulating hormone.
